# Clinical Characteristics and Pharmacological Treatment of Individuals With and Without Intellectual Disability in Pre-trial Assessment—A Population-Based Study

**DOI:** 10.3389/fpsyt.2020.573989

**Published:** 2020-10-23

**Authors:** Hanna Edberg, Qi Chen, Peter Andiné, Henrik Larsson, Tatja Hirvikoski

**Affiliations:** ^1^Department of Women's and Children's Health, Paediatric Neuropsychiatry Unit, Centre for Neurodevelopmental Disorders at Karolinska Institute (KIND), Karolinska Institute, Stockholm, Sweden; ^2^Northern Stockholm Psychiatric Clinic, Region Stockholm, Stockholm, Sweden; ^3^Forensic Psychiatric Clinic, Region Stockholm, Stockholm, Sweden; ^4^Center for Psychiatric Research, Region Stockholm, Stockholm, Sweden; ^5^Department of Medical Epidemiology and Biostatistics, Karolinska Institute, Stockholm, Sweden; ^6^Department of Psychiatry and Neurochemistry, Centre for Ethics, Law and Mental Health (CELAM), Institute of Neuroscience and Physiology, Sahlgrenska Academy, University of Gothenburg, Gothenburg, Sweden; ^7^Forensic Psychiatric Clinic, Sahlgrenska University Hospital, Gothenburg, Sweden; ^8^Department of Forensic Psychiatry, National Board of Forensic Medicine, Gothenburg, Sweden; ^9^School of Medical Sciences, Örebro University, Örebro, Sweden; ^10^Habilitation and Health, Region Stockholm, Stockholm, Sweden

**Keywords:** intellectual disability (ID), forensic psychiatric assessment, neurodevelopmental disorders, pre-trial assessment, mental retardation, forensic psychiatry

## Abstract

**Background:** The current lack of knowledge about intellectual disability (ID) in forensic psychiatric contexts can compromise the legal certainty of these individuals during the medico-legal process. To address ambiguous results in previous literature, the aim of the current study was to estimate the prevalence of ID in a pre-trial forensic psychiatric settings. Moreover, as little is known about the characteristics of offenders with ID, we conducted a clinical characterization of individuals with and without ID being subject to forensic psychiatric assessment.

**Methods:** Using data from several Swedish national registers, we conducted a population-based retrospective observational study on 8,442 individuals being subject to pre-trial forensic psychiatric assessments in Sweden in 1997–2013. We performed univariate analyses to compare the characteristics of individuals with (*n* = 537) and without ID (*n* = 7,905).

**Results:** The prevalence of ID was 6.4% in the Swedish pre-trial forensic psychiatric context during the observational period. Compared with individuals without ID, individuals with ID were younger at the time of assessment, had a lower educational level, and had less frequently started families. ID was associated with lower frequency of diagnosed psychotic and bipolar disorders. However, a similar prescription rate of antipsychotics, and a comparable rate of previous inpatient care was observed among individuals with and without ID. Individuals with ID had more often been prescribed anti-libidinal treatments often used for treating sexual disorders, although did not present a higher prevalence of sexual disorder.

**Conclusions:** The prevalence of ID among pre-trial individuals being subject to forensic psychiatric assessment was more than twice as high as assumed in the general population. Our results suggest that individuals with ID received pharmacotherapy without clear indication. Remaining challenges in the clinical management of individuals with ID were indicated by the discrepancy between the occurrence of psychiatric diagnoses, pharmacological treatment patterns, and rates of inpatient care.

## Introduction

Intellectual disability (ID) is characterized by early onset significant impairment of intellectual functioning and adaptive behaviors (1, 2). ID is assessed using standardized, normed IQ test batteries (3), together with assessment of adaptive behaviors. Based on a normal distribution of intellectual ability, ID corresponds to an intelligence quotient (IQ-score) of more than two standard deviations below the population mean, which applies to ~2.5% of the population. Meta-analyses based on data from different countries, including Sweden, have shown that the prevalence of diagnosed ID in the general population is about 1% (4, 5). ID is in many aspects a risk factor for negative long-term outcomes, such as limited access to healthcare and reduced life expectancy (6–8), and for a subgroup of the population, plausibly criminal behavior (9).

The proportion of individuals with ID among criminal offenders has been difficult to determine, owing to different study settings and different methods used to identify ID (10–19). Differences in mental health legislation between countries and diverse social policy decisions, such as means taken to deinstitutionalize individuals with ID, have also influenced crime rates (20–22). Trying to establish the prevalence of ID among offenders, a variety of study methods have been used, including birth cohorts (23) and cross-sectional studies in different cohorts, pre- and post-trial (11, 14, 17, 24), presenting diverging results. Systematic reviews have suggested a prevalence of ID ranging from 0.5 to 1.5% (25) to as high as 7–10% (19) in prison populations. Previous studies on large Swedish cohorts have indicated that individuals with impaired intellectual ability have a significantly higher risk of criminal and violent behavior than the average population (9, 23). These studies have however applied a less distinctive definition of intellectual disability than current practices, including borderline ID (intelligence quotient 70–85).

There are few studies on the characteristics of criminal offenders with ID, and most are generally conducted on individuals whom have been sentenced to incarceration. However, most developed countries have jurisdiction regarding offenders with mental illness and intellectual disability, where individuals deemed legally incompetent or unfit to stand trial are diverted to secure hospitals instead of being imprisoned (26). Bearing the risk of highly selected samples in mind, previous studies have suggested that offenders with ID are younger, and psychosocially at more of a disadvantage in regards to their familial and housing situations, qualifications, and employment, than non-ID offenders (27–31).

In the general population, psychotic disorders are more common among ID than non-ID individuals, and affective disorders are slightly overrepresented (32–36). However, studies on pharmacological treatment among individuals with ID suggest that prevalence of psychotropic medication, especially antipsychotics, far exceed the prevalence of diagnosed mental illness (37–40).

Focusing on offender populations, previous studies have suggested a higher rate of psychiatric comorbidity such as anxiety, personality disorders, attention-deficit hyperactivity disorder (ADHD), and psychosis among individuals with ID compared to non-ID individuals (27, 28, 41, 42), as well as significantly higher rates of behavioral and conduct disorders (43). However, looking more specifically at forensic psychiatric populations, psychotic disorders may not be overrepresented in individuals with ID (29, 44).

According to The Swedish Penal Code, individuals with a severe mental disorder who commit serious criminal offenses should preferably be ordered to other sentences than prison, mainly forensic psychiatric care (45). Therefore, when indicated, the court requests a forensic psychiatric assessment to determine whether an offender suffers from a severe mental disorder. Among disorders commonly considered as severe mental disorders are psychotic disorders; severe depression with suicidal behavior; severe personality disorder or other mental disorder with recurrent episodes of psychotic behavior, marked compulsiveness, and/or decreased psychosocial functioning; severe dementia; intellectual disability with psychotic symptoms and/or severely reduced psychosocial functioning and severe brain damage. A forensic psychiatric assessment lasts for about 4 weeks and consists of a thorough examination by forensic psychiatrists, psychologists, social workers, and nursing staff. Each year, ~500 offenders undergo forensic psychiatric assessment in Sweden and approximately half of these individuals are sentenced to forensic psychiatric care (46).

The aim of the current study was to estimate the prevalence of ID in the pre-trial forensic psychiatric context and characterize individuals with ID, in comparison to individuals without ID, regarding sociodemographic and domestic factors, psychiatric comorbidity, history of psychiatric inpatient care, and pharmacological treatment.

## Materials and Methods

### Study Design

We conducted a population-based retrospective observational study, including all individuals who underwent forensic psychiatric assessment in Sweden between January 1, 1997 and May 30, 2013 (*n* = 8,442).

### Study Setting

Data from the forensic psychiatric assessments is registered in the Central Archive of the National Board of Forensic Medicine. The archive provided information about ID and concurrent diagnoses. The data was linked to national population-based registers. Data on socioeconomic status and domestic factors were extracted from a longitudinal integration database for health insurance and labor market studies covering all Swedish residents ≥16 years of age (47). The Multi-Generation Register enabled identification of parents. The Total Population Register provided information on sex, birth year, and migration status (48). Psychiatric diagnoses and psychiatric inpatient care prior to the forensic psychiatric assessment were obtained from the National Patient Register (NPR). The diagnoses in NPR are coded according to the International Classification of Diseases (ICD) (2, 49). The Prescribed Drug Register (PDR) covers prescription medication that has been dispensed from Swedish pharmacies since July 2005 (50).

### Ascertainment of ID Diagnosis

The categorization of ID and non-ID individuals was based upon the forensic psychiatric assessment. The forensic psychiatric assessment is standardized and team-based. The individual is thoroughly evaluated including a medical-psychiatric assessment with standardized diagnostic tests and examinations, a psychological assessment of intellectual and adaptive functioning including Wechsler Adult Intelligence Scale (3) when applicable, a comprehensive compilation of the individuals' social circumstances made by forensic social workers, and 24-h observations by nursing staff. The diagnostic classification system used during our study period was the Diagnostic and Statistical Manual of Mental Disorders, fourth version (DSM-IV) (51). The diagnostic term for ID in DSM-IV was mental retardation. We identified all individuals with a DSM-IV diagnosis of mental retardation (317, 318.0, 318.1, 318.2, and 319). Information regarding ID prior to the forensic psychiatric assessment was obtained from the NPR. The NPR applies diagnostic codes according to the international classification system (ICD). Codes for ID in ICD-8 (311, 312, 313, 314, and 315) and in ICD-9 (317, 318, and 319) were converted to corresponding ICD-10 diagnoses using a conversion instrument provided by the Swedish National Board of Health and Welfare. Codes for ID in ICD-10 were F70, F71, F72, F73, F78, and F79 (2).

### Sociodemographic and Domestic Factors

#### Classification of Immigration Status

We defined three categories of immigration status: born outside Sweden, born in Sweden with one, or both, parents born outside Sweden.

#### Classification of Socioeconomic Status

We used parental education level as a proxy for socioeconomic status. For individuals ≥ 20 years of age, we also included individual education level. Education was divided into three levels, <9 years (low), 9 years (medium), and > 9 years (high). Income levels were assessed for individuals ≥ 18 years of age, by studying risk of poverty (defined by Statistics Sweden as a household income of <60% of median national income). In order to avoid floor effects in the statistical analyses, we also analyzed “low income/poverty,” defined as a household income of <20% of the median national income.

#### Classification of Domestic Factors

We registered if the individual was married, had children, and whether or not his/her parents were still alive.

### Clinical History of Psychiatric Diagnoses and Treatment

#### Classification of Psychiatric Diagnoses Prior to the Forensic Psychiatric Assessment

Psychiatric diagnoses were categorized according to the international classification system (ICD). Codes in ICD-8 and ICD-9 were converted to corresponding ICD-10 diagnoses as described above. Diagnoses were grouped into 18 categories, namely schizophrenia (F20), other psychotic disorders (F21–29), depressive disorders (F32, F33, F34, F38, and F39), bipolar disorders (F31, F31), antisocial personality disorders (F60.2), borderline personality disorders (F60.3), other personality disorders (F60.0, F60.1, F60.4, F60.5, F60.6, F60.7, F60.8, F60.9, and F61.9), ADHD (F90), autism spectrum disorders (ASD) (F84), disorders due to alcohol use (F10), disorders due to drug use (F11–F19), phobic disorders (F40), anxiety disorders (F41), obsessive-compulsive disorders (OCD) (F42), stress or adjustment disorders (F43), impulse control disorders (F63), sexual disorders (F65, F66.2), and epilepsy (G40, G41). A pre-existing diagnosis of ID was classified according to ICD as described in section Ascertainment of ID diagnosis.

#### Psychiatric Inpatient Care Prior to the Assessment

Data from the NPR regarding previous psychiatric inpatient care was defined at three levels (none, once, or more than once).

#### Suicide Attempt Prior to the Assessment

We used ICD-10-codes for “intentional self-harm” (X60-X84) to categorize suicide attempts. Codes in ICD-8 and ICD-9 were converted to corresponding ICD-10 diagnoses as described above.

#### Pharmacological Treatment Prior to the Assessment

Pharmacological treatment was defined as prescription medications collected from the pharmacy within 1 year prior to the forensic psychiatric assessment. The substances were grouped into 11 categories based on the Anatomical Therapeutic Chemical (ATC)-codes: antipsychotics (N05AA, N05AB, N05AC, N05AD, N05AE, N05AF, N05AG, N05AH, and N05AX), antidepressants (N06AA, N06AB, N06AC, N06AD, N06AE, N06AF, N06AG, and N06AX), lithium (N05AN), anti-epileptics (N03AA, N03AB, N03AC, N03AD, N03AF, N03AG, N03AX14, and N03AX09), anxiolytics/sedatives (non-benzodiazepines) (N05BB, N05BE N05CH, and N05CM), ADHD-medication (N06BA01, N06BA02, N06BA03, N06BA04, N06BA05, N06BA06, N06BA09, N06BA10, N06BA12, and N06BA13), anticholinergics (N04AA01, N04AA02), and drugs aimed at treating substance abuse (N07BC01, N07BC51, N07BC02, N07BB01, N07BB03, N07BB04, and N07BB05). To analyze treatment of sexual disorders, we created the category hormonal treatment, (G03HA01, L02AE) including a selection of progestogens, anti-androgens, and gonadotropin-releasing hormone (GnRH) analogs, based upon current guidelines for treatment of sexual disorders (52). We chose to separate benzodiazepines (N05BA, N05CD, and N03AE) from benzodiazepine-like sedatives (zolpidem and zopiclone) (N05CF), since use of benzodiazepines may affect the risk of violent crime (53).

### Psychiatric Diagnoses According to Forensic Psychiatric Assessment

Based on current clinical practice, the psychiatric diagnoses established during the forensic psychiatric assessment were categorized according to DSM-IV. Diagnoses were grouped into the same categories as for lifetime psychiatric comorbidity with the exception of phobic disorders, anxiety disorders, and epilepsy, since these are seldom of importance in a forensic psychiatric assessment and therefore often not assessed. The following diagnostic codes were used: schizophrenia (295 except 295.70), other psychotic disorders (295.70, 297, and 298), depressive disorders (296.2, 296.3, 296.9, 311, and 300.4), bipolar disorders (296.0, 296.4, 296.5, 296.6, 296.7, 296.8, and 313.13), antisocial personality disorders (301.7), borderline personality disorders (301.83), other personality disorders (301 except 301.7 and 301.83), ADHD (314), autism spectrum disorders (ASD) (299), disorders due to alcohol use (291, 303.00, 303.90, and 305.00), disorders due to drug use (292, 304, 305 except 305.00), obsessive-compulsive disorders (OCD) (300.3), stress or adjustment disorders (308.3, 309), impulse control disorders (312.3), and sexual disorders (302 except gender identity disorders 302.6 and 302.85).

### Statistical Analyses

Prevalence of ID during the observational period was calculated as the proportion of cases of ID in the entire study population. Comparison between individuals with and without ID was made using Mann–Whitney *U*-test for continuous variables and Chi-square tests for categorical variables. The statistical tests were two-sided and the alpha level was set at *p* < 0.05. SAS version 9.4 was used to generate analytic datasets and R version 3.5 was used for data analyses.

## Results

**Figure 1:**
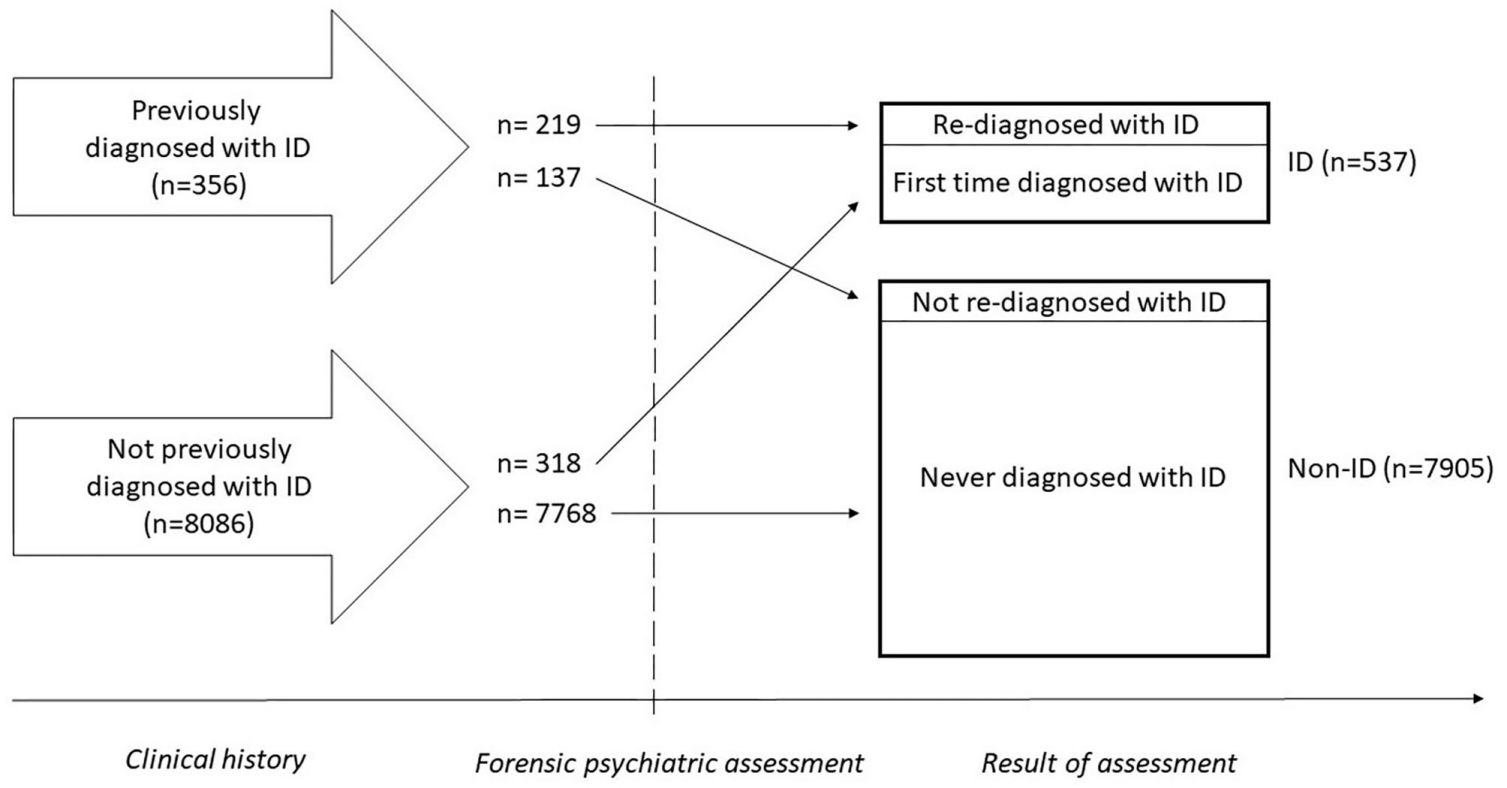
Timeline illustrating diagnostic status from clinical history to forensic psychiatric assessment.

Among 8,442 individuals being subject to a pre-trial forensic psychiatric assessment in Sweden during the years 1997–2013, 537 were diagnosed with ID. Thus, the prevalence of ID was 6.4%. Out of these 537 individuals, 434 (80.8%) were diagnosed with mild ID, 61 (11.4%) with moderate or severe ID, and 42 (7.8%) with ID not otherwise specified. A total of 318 (59.2%) had not previously been diagnosed with ID and thus got their diagnosis of ID for the first time during the assessment (Figure 1).

### Sociodemographic Factors

The sociodemographic factors among individuals with and without ID, respectively, and statistics for group comparisons are depicted in Table 1. Compared with individuals without ID, individuals with ID were on average seven years younger than their non-ID counterparts (*p* < 0.001) and a greater number had living parents (79.7 vs. 69%, *p* < 0.001) at the time of forensic psychiatric assessment. Individuals with ID had less often formed a family including marriage (4.7 vs. 10.8%, *p* < 0.001) and children (24.2 vs. 50.7%, *p* < 0.001). ID individuals were less often born outside Sweden (25.3 vs. 32.7%, *p* < 0.001).

**Table 1:** Sociodemographic characteristics of ID and non-ID individuals subject to forensic psychiatric assessment during 1997–2013.

	**ID^a^*n* = 537**	**Non-ID^a^*n* = 7,905**	**Statistics^b^**	***P*-value**
**Demographic factors**				
Median (IQR) age, years	27 (21–38)	35 (26–44)	2,712,600^c^	<0.001
Male, *n* (%)	456 (84.9)	6,942 (87.8)	3.643	0.056
**Immigration status**, ***n*** **(%)**				
Missing	1 (0.2)	3 (0.0)		
Born outside Swe	136 (25.3)	2583 (32.7)	11.965	0.001
Born in Swe	400 (74.3)	5319 (67.3)		
**Parental immigration status^d^, ***n*** (%)**				
0 parent born outside Swe	296 (74.0)	3,831 (72.0)		
1 parent born outside Swe	58 (14.5)	889 (16.7)	2.303	0.316
2 parents born outside Swe	45 (11.2)	508 (9.6)		
Unknown	1 (0.2)	91 (1.7)		
**Domestic factors**, ***n*** **(%)**				
Married	25 (4.7)	855 (10.8)	19.783	<0.001
Children	130 (24.2)	4007 (50.7)	140.042	<0.001
Parent(s) alive	428 (79.7)	5452 (69.0)	26.898	<0.001
**Parental education level**, ***n*** **(%)**				
Low (<9 years)	112 (20.9)	1,128 (14.3)		
Medium (=9 years)	70 (13.0)	480 (6.1)	41.444	<0.001
High (>9 years)	224 (41.7)	3,598 (45.5)		
Missing	131 (24.4)	2,699 (34.1)		
**Individuals** **≥** **18 years of age**	**ID** ***n*** **=** **511**	**Non-ID** ***n*** **=** **7,768**	**Statistics**	***P*****-value**
**Risk of poverty**, ***n*** **(%) (household income)**				
No	233 (45.6)	3,738 (48.1)		
Yes	276 (54.0)	3,898 (50.2)	1.802	0.179
Missing	2 (0.4)	132 (1.7)		
**Low income/Poverty**, ***n*** **(%) (household income)**				
No	484 (94.7)	6,963 (89.6)		
Yes	25 (4.9)	673 (8.7)	8.781	0.003
Missing	2 (0.4)	132 (1.7)		
**Individuals** **≥** **20 years of age**	**ID** ***n*** **=** **461**	**Non-ID** ***n*** **=** **7,396**	**Statistics**	***P*****-value**
**Individual educational level**, ***n*** **(%)**				
Low (<9 years)	72 (15.6)	644 (8.7)		
Medium (=9 years)	206 (44.7)	2,445 (33.1)	120.449	<0.001
High (>9 years)	110 (23.9)	3,952 (53.4)		
Missing	73 (15.8)	355 (4.8)		

*IQR, Interquartile range; Swe, Sweden*.

a *According to the forensic psychiatric assessment*.

b *X^2^ for Chi-square test unless otherwise specified*.

c *W for Mann–Whitney U Test*.

d *Only individuals born in Sweden*.

The ID group had lower educational level than non-ID group: twice as often <9 years of education (15.6 vs. 8.7%, *p* < 0.001) and half as often more than 9 years of education (23.9 vs. 53.4%, *p* < 0.001). The same pattern was seen regarding the educational level of their parents, although the parental differences were not as pronounced.

Compared to the national population levels, our study population of individuals being subject to forensic psychiatric assessment was economically challenged. About 50% were at risk of poverty (i.e., a household income of <60% of median national income according to Statistics Sweden's definition), compared to about 15% in the general population (54). There was, however, no significant difference regarding risk of poverty between ID and non-ID groups. Moreover, individuals with ID had lower risk of being in the “low income/poverty” category (4.9 vs. 8.7%, *p* < 0.001).

### Clinical History Prior to Forensic Psychiatric Assessment

The descriptive statistics regarding clinical history are shown in Table 2. A total of 356 individuals already had a diagnosis of ID before entering the forensic psychiatric assessment, and 219 of these were re-diagnosed with ID during the assessment.

**Table 2:** Psychiatric diagnoses and psychiatric inpatient care prior to the forensic psychiatric assessment.

	**ID^a^*n* = 537**	**Non-ID^a^*n* = 7,905**	**Statistics, *X*^**2**^**	***P*-value**
ID diagnosis before forensic psychiatric assessment^b^, *n* (%)	219 (40.8)	137 (1.7)	1,888.617	<0.001
**PSYCHIATRIC COMORBIDITY**, ***n*** **(%)**				
**Psychoses**				
Schizophrenia	36 (6.7)	1,052 (13.3)	18.951	<0.001
Other psychotic disorders	92 (17.1)	2,159 (27.3)	26.129	<0.001
**Mood disorders**				
Depressive disorder	99 (18.4)	1,675 (21.2)	2.134	0.144
Bipolar disorder	13 (2.4)	538 (6.8)	15.137	<0.001
**Personality disorders (pd)**				
Antisocial pd	11 (2.0)	324 (4.1)	5.022	0.025
Borderline pd	23 (4.3)	520 (6.6)	4.028	0.045
Other pd	80 (14.9)	1,649 (20.9)	10.614	0.001
**Neurodevelopmental disorders**				
ADHD	72 (13.4)	510 (6.5)	36.831	<0.001
ASD	63 (11.7)	523 (6.6)	19.588	<0.001
**Disorders due to substance use**				
Disorders d/t alcohol use	120 (22.3)	2,103 (26.6)	4.481	0.034
Disorders d/t drug use	99 (18.4)	2,587 (32.7)	46.678	<0.001
**Anxiety disorders**				
Phobic disorders	11 (2.0)	169 (2.1)	0.000	1.000
Anxiety disorders	83 (15.5)	1,123 (14.2)	0.544	0.461
OCD	18 (3.4)	181 (2.3)	2.025	0.155
Stress or adjustment disorder	89 (16.6)	1430 (18.1)	0.684	0.408
Impulse control disorder	41 (7.6)	194 (2.5)	47.978	<0.001
Sexual disorder	4 (0.7)	35 (0.4)	0.998	0.291
**Other relevant comorbidity**, ***n*** **(%)**				
Epilepsy	50 (9.3)	288 (3.6)	40.565	<0.001
**Psychiatric inpatient care**, ***n*** **(%)**				
Never	185 (34.5)	2,547 (32.2)		
Once	74 (13.8)	1,143 (14.5)	1.161	0.560
More than once	278 (51.8)	4,215 (53.3)		
Suicide attempt	89 (16.6)	1,629 (20.6)	4.802	0.028
	**ID** ***n*** **=** **238**	**Non-ID** ***n*** **=** **2,878**	**Statistics**, ***X***^**2**^	***P*****-value**
**Pharmacological treatment^c^, ***n*** (%)**				
Antipsychotics	76 (31.9)	847 (29.4)	0.546	0.460
Antidepressants	67 (28.2)	880 (30.6)	0.502	0.479
Lithium	6 (2.5)	87 (3.0)	0.057	0.811
Antiepileptics	42 (17.6)	265 (9.2)	16.690	<0.001
Benzodiazepines (bzd)	45 (18.9)	581 (20.2)	0.152	0.697
Sedatives (bzd-like)	36 (15.1)	611 (21.2)	4.614	0.032
Anxiolytics/sedatives (non-bzd)	57 (23.9)	644 (22.4)	0.228	0.633
Anti-libidinal treatment	5 (2.1)	4 (0.1)	29.378	<0.001
ADHD medication	22 (9.2)	180 (6.3)	2.766	0.096
Anticholinergics	12 (5.0)	134 (4.7)	0.012	0.911
Anti-dependence drugs	12 (5.0)	164 (5.7)	0.076	0.783

*ADHD, attention-deficit hyperactivity disorder; ASD, autism spectrum disorder; OCD, obsessive compulsive disorder*.

a *According to the forensic psychiatric assessment*.

b *Individuals having a registered diagnosis of ID before entering the forensic psychiatric assessment*.

c *Prescription medication collected from the pharmacy within one year prior to the forensic psychiatric assessment*.

Individuals diagnosed with ID during the forensic psychiatric assessment (*n* = 537) had more often than their non-ID counterparts a clinical history of concurrent neurodevelopmental disorder (ADHD: 13.4 vs. 6.5%; ASD: 11.7 vs. 6.6%, *p* < 0.001) and impulse control disorder (7.6 vs. 2.5%, *p* < 0.001). As for the occurrence of depressive disorders and anxiety disorders, no significant differences were observed.

On the contrary, individuals with ID were less frequently than non-ID individuals diagnosed with psychotic disorders (schizophrenia: 6.7 vs. 13.3%; other psychotic disorders: 17.1 vs. 27.3%, *p* < 0.001), bipolar disorder (2.4 vs. 6.8%, *p* < 0.001), personality disorders (antisocial personality disorder: 2.0 vs. 4.1%, *p* = 0.025; borderline personality disorder: 4.3 vs. 6.6%, *p* = 0.045; other personality disorder: 14.9 vs. 20.9%, *p* = 0.001), and disorders due to drug use (18.4 vs. 32.7%, *p* < 0.001). However, there was no significant difference in prescriptions of antipsychotics, lithium, or medication for addiction disorders. A discrepancy between diagnosis vs. treatment was similarly observed regarding sexual disorders: while there was no significant between-group difference in the incidence of sexual disorders, treatment with anti-libidinal medication was more common among individuals with ID (2.1 vs. 0.1%, *p* < 0.001). Individuals with ID were almost twice as often treated with antiepileptics (17.6 vs. 9.2%, *p* < 0.001) corresponding to a higher prevalence of epilepsy (9.3 vs. 3.6%, *p* < 0.001). Non-ID individuals had to a greater extent than individuals with ID a history of suicide attempt (20.6 vs. 16.6%, *p* = 0.028), while there was no significant difference between ID and non-ID individuals regarding previous psychiatric inpatient care.

#### ID Diagnostic Status in Clinical History and During the Forensic Psychiatric Assessment

As previously mentioned, 356 individuals had been diagnosed with ID before the forensic psychiatric assessment. Out of these, 137 were not re-diagnosed with ID during the assessment. Comparing these 137 individuals with the 7,768 individuals who were never diagnosed with ID, a significantly higher incidence of ASD (27.0 vs. 7.0%, *p* < 0.001) and ADHD (19.0 vs. 6.1%, *p* < 0.001) was observed during the forensic psychiatric assessment (Table 3).

**Table 3:** Psychiatric diagnoses during the forensic psychiatric assessment among individuals with a history of ID, whom were not re-diagnosed with ID during the assessment, compared to individuals who never had an ID diagnosis.

	**Previous diagnosis of ID, not re-diagnosed with ID *n* = 137**	**Never diagnosed with ID *n* = 7,768**	**Statistics, *X*^**2**^**	***P*-value**
**DIAGNOSES ACCORDING TO FORENSIC PSYCHIATRIC ASSESSMENT**, ***n*** **(%)**				
**Psychoses**				
Schizophrenia	22 (16.1)	1,006 (13.0)	0.891	0.345
Other psychotic disorders	11 (8.0)	1,318 (17.0)	7.064	0.008
**Mood disorders**				
Depressive disorder	6 (4.4)	775 (10.0)	4.129	0.042
Bipolar disorder	3 (2.2)	261 (3.4)	0.266	0.606
**Personality disorders (pd)**				
Antisocial pd	16 (11.7)	868 (11.2)	0.002	0.961
Borderline pd	13 (9.5)	548 (7.1)	0.869	0.351
Other pd	28 (20.4)	1,802 (23.2)	0.432	0.511
**Additional neurodevelopmental disorders**				
ADHD	26 (19.0)	473 (6.1)	35.669	<0.001
ASD	37 (27.0)	545 (7.0)	75.983	<0.001
**Disorders due to substance use**				
Disorders d/t alcohol use	36 (26.3)	1,976 (25.4)	0.016	0.901
Disorders d/t drug use	29 (21.2)	2,538 (32.7)	7.610	0.006
OCD	5 (3.6)	77 (1.0)	6.859	0.009
Stress or adjustment disorder	4 (2.9)	825 (10.6)	7.704	0.006
Impulse control disorder	11 (8.0)	240 (3.1)	9.138	0.003
Sexual disorder	3 (2.2)	205 (2.6)	0.003	0.955

*ADHD, attention-deficit hyperactivity disorder; ASD, autism spectrum disorder; OCD, obsessive compulsive disorder*.

We also compared those who received an ID diagnosis for the first time during the forensic psychiatric assessment (*n* = 318, 59.2% out of the total 537 individuals with ID) with those who had a diagnosis already upon entering the assessment and were re-diagnosed during the assessment (*n* = 219) with regard to clinical history and background variables (Table 4). The individuals who had not been previously recognized as having ID were more often male (89.0 vs. 79.0%, *p* = 0.003), married (5.3 vs. 3.7%, *p* < 0.001) and had more often children (30.5 vs. 15.1%, *p* < 0.001). They had a lower incidence of schizophrenia (3.5 vs. 11.4%, *p* = 0.001), other psychotic disorders (10.7 vs. 26.5%, *p* < 0.001), depressive disorder (15.4 vs. 22.8%, *p* = 0.039), ADHD (7.2 vs. 22.4%, *p* < 0.001), ASD (5.3 vs. 21.0%, *p* < 0.001), stress or adjustment disorder (11.0 vs. 24.7%, *p* < 0.001), impulse control disorder (4.1 vs. 12.8%, *p* < 0.001), and epilepsy (5.7 vs. 14.6%, *p* < 0.001) in their clinical history. They had to a lesser extent been subject to psychiatric inpatient care (37.3 vs. 72.1%, *p* < 0.001) or made a suicide attempt (12.3 vs. 22.8%, *p* = 0.002). Previously un-recognized ID was not associated with immigration status of the individuals with ID or their parents (*p* > 0.05).

**Table 4:** Individuals diagnosed with ID for the first time at the forensic psychiatric assessment (*n* = 318), compared to individuals who already had ID diagnosis and were re-diagnosed with ID (*n* = 219), with regard to background variables and clinical history.

	**First time diagnosis of ID at assessment *n* = 318**	**Previous diagnosis of ID, re-diagnosed at assessment *n* = 219**	**Statistics^a^**	***P*-value**
**Demographic factors**				
Median (IQR) age, years	28 (22–38)	26 (21–38)	36,437^b^	0.360
Male, *n* (%)	283 (89.0)	173 (79.0)	9.356	0.003
**Immigration status**, ***n*** **(%)**				
Missing	0 (0.0)	1 (0.5)		
Born outside Swe	84 (26.4)	52 (23.7)	0.323	0.570
Born in Swe	234 (73.6)	166 (75.8)		
**Parental immigration status**^c^, ***n*** **(%)**				
0 parent born outside Swe	29 (12.4)	16 (9.6)		
1 parent born outside Swe	35 (15.0)	23 (13.9)	0.873	0.646
2 parents born outside Swe	170 (72.6)	126 (75.9)		
Unknown	0 (0.0)	1 (0.6)		
**Domestic factors**, ***n*** **(%)**				
Married	17 (5.3)	8 (3.7)	0.499	<0.001
Children	97 (30.5)	33 (15.1)	16.008	<0.001
Parent(s) alive	250 (78.6)	178 (81.3)	0.415	0.519
**Parental education level**, ***n*** **(%)**				
Low (<9 years)	66 (20.8)	46 (21.0)		
Medium (=9 years)	44 (13.8)	26 (11.9)		
High (>9 years)	126 (39.6)	98 (44.7)	0.997	0.607
Missing	82 (25.8)	49 (22.4)		
Individuals ≥ 18 years of age	*n* = 306	*n* = 205		
**Risk of poverty**, ***n*** **(%) (household income)**				
No	147 (48.0)	86 (42.0)		
Yes	157 (51.3)	119 (58.0)	1.773	0.183
Missing	2 (0.7)	0 (0.0)		
**Low income/Poverty**, ***n*** **(%) (household income)**				
No	289 (94.4)	195 (95.1)		
Yes	15 (4.9)	10 (4.9)	1.000	<.001
Missing	2 (0.7)	0 (0.0)		
Individuals ≥ 20 years of age	*n* = 278	*n* = 183		
**Individual educational level**, ***n*** **(%)**				
Low (<9 years)	45 (16.2)	27 (14.8)		
Medium (=9 years)	122 (43.9)	84 (45.9)	5.704	0.058
High (>9 years)	80 (28.8)	30 (16.4)		
Missing	31 (11.2)	42 (23.0)		
**PSYCHIATRIC COMORBIDITY IN CLINICAL HISTORY**, ***n*** **(%)**				
**Psychoses**				
Schizophrenia	11 (3.5)	25 (11.4)	11.885	0.001
Other psychotic disorders	34 (10.7)	58 (26.5)	21.683	<0.001
**Mood disorders**				
Depressive disorder	49 (15.4)	50 (22.8)	4.270	0.039
Bipolar disorder	5 (1.6)	8 (3.7)	1.577	0.209
**Personality disorders (pd)**				
Antisocial pd	6 (1.9)	5 (2.3)	<0.001	0.993
Borderline pd	10 (3.1)	13 (5.9)	1.831	0.176
Other pd	39 (12.3)	41 (18.7)	3.771	0.052
**Additional neurodevelopmental disorders**				
ADHD	23 (7.2)	49 (22.4)	24.322	<0.001
ASD	17 (5.3)	46 (21.0)	29.213	<0.001
**Disorders due to substance use**				
Disorders d/t alcohol use	66 (20.8)	54 (24.7)	0.925	0.336
Disorders d/t drug use	55 (17.3)	44 (20.1)	0.501	0.479
**Other psychiatric disorders**				
OCD	6 (1.9)	12 (5.5)	4.118	0.042
Stress or adjustment disorder	35 (11.0)	54 (24.7)	16.506	<0.001
Impulse control disorder	13 (4.1)	28 (12.8)	12.705	<0.001
Sexual disorder	0 (0.0)	4 (1.8)	3.642	0.056
**Other relevant comorbidity**, ***n*** **(%)**				
Epilepsy	18 (5.7)	32 (14.6)	11.269	0.001
**Psychiatric inpatient care**, ***n*** **(%)**				
Never	160 (50.3)	25 (11.4)		
Once	38 (11.9)	36 (16.4)	88.519	<0.001
More than once	120 (37.7)	158 (72.1)		
Suicide attempt	39 (12.3)	50 (22.8)	9.723	0.002

*IQR, Interquartile range; Swe, Sweden; ADHD, attention-deficit hyperactivity disorder; ASD, autism spectrum disorder; OCD, obsessive compulsive disorder*.

a *X^2^ for Chi-square test unless otherwise specified*.

b *W for Mann–Whitney U Test*.

c *only individuals born in Sweden*.

### Psychiatric Diagnoses According to Forensic Psychiatric Assessment

Table 5 depicts the descriptive statistics from the forensic psychiatric assessment. Similar to the clinical history, individuals with ID (*n* = 537) had a lower frequency of psychotic disorders, mood disorders, personality disorders, and disorders due to drug use. The difference in incidence of sexual disorders did not reach statistical significance (ID: 4.1%; non-ID: 2.6%, *p* = 0.060).

**Table 5:** Psychiatric diagnoses according to the forensic psychiatric assessment.

	**ID^a^*n* = 537**	**Non-ID^a^*n* = 7,905**	**Statistics, *X*^**2**^**	***P*-value**
**DIAGNOSES ACCORDING TO FORENSIC PSYCHIATRIC ASSESSMENT**, ***n*** **(%)**				
**Psychoses**				
Schizophrenia	19 (3.5)	1,028 (13.0)	40.609	<0.001
Other psychotic disorders	63 (11.7)	1,329 (16.8)	9.059	0.003
**Mood disorders**				
Depressive disorder	25 (4.7)	781 (9.9)	15.293	<0.001
Bipolar disorder	4 (0.7)	264 (3.3)	10.186	0.001
**Personality disorders (pd)**				
Antisocial pd	30 (5.6)	884 (11.2)	15.737	<0.001
Borderline pd	15 (2.8)	561 (7.1)	13.979	<0.001
Other pd	73 (13.6)	1,830 (23.1)	25.753	<0.001
**Additional neurodevelopmental disorders**				
ADHD	63 (11.7)	499 (6.3)	22.902	<0.001
ASD	93 (17.3)	582 (7.4)	66.407	<0.001
**Disorders due to substance use**				
Disorders d/t alcohol use	120 (22.3)	2,012 (25.5)	2.408	0.121
Disorders d/t drug use	75 (14.0)	2,567 (32.5)	79.239	<0.001
OCD	8 (1.5)	82 (1.0)	0.594	0.441
Stress or adjustment disorder	32 (6.0)	829 (10.5)	10.768	0.001
Impulse control disorder	60 (11.2)	251 (3.2)	88.412	<0.001
Sexual disorder	22 (4.1)	208 (2.6)	3.541	0.060

*ADHD, attention-deficit hyperactivity disorder; ASD, autism spectrum disorder; OCD, obsessive compulsive disorder*.

a *According to the forensic psychiatric assessment*.

## Discussion

ID was overrepresented in this population-based, nation-wide, pre-trial forensic psychiatric assessment cohort compared to the general population. Individuals with ID were younger and less socioeconomically independent than their non-ID counterparts. ID individuals were less often diagnosed with serious psychiatric disorders, but they were as often, or even more often, than non-ID individuals prescribed psychopharmacological treatment, including antipsychotics and anti-libidinal medication. Among individuals who had an ID diagnosis when entering the forensic psychiatric assessment but who were not re-diagnosed with ID during the assessment, a high incidence of other neurodevelopmental disorders (ADHD and ASD) was observed.

### Prevalence of ID in a Population-Based Pre-trial Forensic Psychiatric Cohort

The prevalence of ID in this Swedish nation-wide population-based cohort of 8,442 individuals who had been subject to pre-trial forensic psychiatric assessment was more than twice as high as assumed in the general population based on the normal distribution of IQ (6.4 vs. 2.5%), and more than six times as high as the recorded prevalence of ID in the general population (1.0%) (4, 5). International comparisons of pre-trial populations are challenging, since legislation as well as national guidelines and clinical practices affect the sample selection. In addition, whether or not the psychiatric assessment is mandatory (court ordered) or voluntary will affect representativeness. Nation-wide pre-trial population studies similar to ours, where the psychiatric assessment was court-ordered and refusal rates therefore negligible, in Finland (55) and the Netherlands (43), reported lower prevalence rates of ID (2–3%) compared to our results. Thus, the proportion of individuals with ID in pre-trial forensic psychiatric populations in Sweden was 2- to 3-fold higher than in neighboring European countries. Even though we do not know the exact reasons for this, one possible explanation may be a faster process of deinstitutionalization in Sweden compared to neighboring countries, leading to a complete closure of large institutions for individuals with ID during the 1980–1990s (56) and a relatively large decline in hospital beds in Sweden during 1990–2002, compared to several other European countries (57). Since the decline in inpatient psychiatric care in Sweden was not followed by a corresponding increase in supported housing (ibid.), vulnerable individuals may have lacked supportive environmental factors, thus resulting in increased risk of criminal offenses. However, almost 60% of those who were diagnosed with ID during the forensic psychiatric assessment in the current study were previously un-recognized as having ID. This indicates that individuals with ID are under-diagnosed which in turn can lead to poor adjustment in school, during education and work, all of which are potential risk factors for antisocial and criminal engagement.

The group who received an ID diagnosis for the first time during the forensic psychiatric assessment (59.2% of all diagnosed with ID) had several distinct clinical characteristics. Compared to individuals who were previously identified as ID and re-diagnosed, they had a higher degree of social adaptation (e.g., married and had children), less often psychiatric comorbidity, and had less often been subject to inpatient psychiatric care, which may partly explain why they had not previously been identified as ID. The reason why these individuals were not recognized as having ID at school could not be analyzed within the frameworks of the current study. This is an important topic for future research, given that early identification of ID and individually adjusted support and interventions may be one of the most important measures preventing criminality in this population.

On the other hand, 137 individuals were enrolled in the forensic psychiatric assessment with a previous diagnosis of ID, and were not re-diagnosed with ID during the assessment. We found that these 137 individuals had a considerably higher incidence of ASD and ADHD than individuals who had never received an ID diagnosis. ID in the clinical history of these cases might therefore have been diagnosed on inadequate clinical grounds. Discontinuance of an ID diagnosis can however also reflect the fact that ID is not an absolute, invariant trait (58–60). Our findings emphasize the need of standardized assessment (61) of individuals with ID in forensic settings, in order to secure legal certainty, and to provide adequate support and treatment.

### Sociodemographic Factors

In line with previous studies, individuals with ID were younger than non-ID individuals, had less often founded a family of their own, and had lower educational levels (27, 29, 43, 55). Our results also suggest that the parents of individuals with ID more often had low educational levels (<9 years of education) than parents of non-ID individuals. In this cohort, pre-trial defendants being subject to forensic psychiatric assessment had an overall low socioeconomic standard. More than half of the individuals in the study cohort were classified as in risk of poverty according to international definitions (62).

Early intervention programs in educational settings and continued support during sensitive life periods such as during transition to adulthood, with increased focus on community inclusion and meaningful occupational activities for individuals with ID can serve as preventive measures and ought to be on the agenda for national policy makers. Financial provision such as income support and secure housing arrangements is a crucial part of socioeconomic stability. In this particular context, and in light of individuals with ID acting on a restricted social arena with fewer significant relationships (63), an improved social network should also be mentioned as a way of achieving enhanced societal inclusion, possibly serving as a protective factor against criminality. Finally, the need for information among family members and significant others has to be met in order for them to identify risk factors and undertake protective measures on familial and individual levels.

### Psychiatric Diagnoses and Treatment

In line with previous findings (29, 37, 39, 43, 44, 64, 65) individuals with ID in our study were less often than their non-ID counterparts diagnosed with serious mental disorders such as psychotic or bipolar disorders. They were however prescribed antipsychotic medication to same extent. Our clinical experience is that individuals with ID often receive antipsychotic medication in order to treat behavioral problems. Similar off-label prescription is troublesome since a review of 56 studies showed no positive effect of psychotropic medication, including antipsychotics, merely treating the challenging behaviors of individuals with ID (66). Antipsychotics are potent substances with potentially severe side effects and our findings emphasize the need for accurate, structured diagnostic assessment, correct treatment indication, and careful follow-up for individuals with ID.

Similarly, we found a higher incidence of anti-libidinal treatment among ID individuals, in absence of a higher prevalence of sexual disorders. Studies on anti-libidinal treatment in ID populations are scarce, but our findings were supported by a previous study in a forensic psychiatric cohort (67). Problematic sexual behavior might not always reach the diagnostic criteria for sexual disorders, but might nevertheless explain findings from recent studies suggesting that ID is overrepresented among at least a subgroup of criminal offenders, such as sexual offenders (30, 31). A possible increased risk of sexual disorders or sexually troublesome behavior among a subgroup of individuals with ID is not only an important scope for future research, but also vital to address early in the clinical and educational settings meeting children and adolescents with ID.

In regard to mood disorders, our findings are consistent with previous data showing no difference in the incidence of depression or in the prescription of antidepressants between ID and non-ID groups (32, 68). As for previous psychiatric inpatient care, there were no significant differences between ID and non-ID groups.

### Strengths and Limitations

Using data from large national registers, we were able to perform a total population study of almost 8,500 individuals being subject to pre-trial forensic psychiatric assessment in Sweden during a period of 17 years. Register data carries the advantage of extensive information. However, inherent shortcomings in the registers might be difficult to rectify, such as incomplete coverage in the registers regarding information about educational levels.

Even though the forensic assessment is thorough, the main purpose is establishment of the concept severe mental disorder. The diagnostic reliability of psychiatric diagnoses might consequently be influenced in a negative manner, resulting in a false negative outcome. This might be especially true concerning diagnoses not considered as severe mental disorders in the context of the forensic psychiatric assessment, such as anxiety disorders. However, we also obtained diagnostic data from the National Patient Register and the diagnostic findings were consistent, thus increasing internal validity.

As for pharmacological treatment, by studying the prevalence of diagnoses and prescription patterns, we are able to identify correlations based upon probable treatment indication. However, a majority of pharmaceuticals have more than one treatment indication. Worth mentioning is that anti-libidinal treatment (L02AE) can be prescribed for prostate cancer and anti-epileptics as mood stabilizers, as well as epilepsy treatments. Furthermore, the Prescribed Drug Register only covers prescription medications dispensed since 2005, while our study period lasted from 1997 to 2013.

## Conclusion

Presenting total population data on offenders with ID being subject to pre-trial forensic psychiatric assessment during a 17-year period in Sweden, these results contribute to the knowledge of prevalence and characteristics of ID offenders. Diagnostic data and pharmacological prescription patterns revealed an imbalance between diagnoses and treatments among individuals with ID in their clinical history, suggesting the possibility of off-label treatments. For example, our findings indicated that individuals with ID were prescribed antihormonal treatment, a potential treatment of paraphilias, and sexual offending behavior, to a greater extent than their non-ID counterparts, in absence of a higher incidence of sexual disorders. However, our study included few cases and further research is needed to more comprehensively address this issue. Studies of pharmacological treatment in a forensic psychiatric context and effect of forensic psychiatric care on criminal recidivism, would be of interest to increase knowledge on best practices for individuals with ID in a forensic psychiatric settings.

The study shows that there are many similarities but also important differences between offenders with and without ID, as well as within the ID group, highlighting the importance of adequate clinical assessment. The fact that more than half of those who received an ID diagnosis during the forensic psychiatric assessment had not previously been diagnosed with ID, suggests that these diagnoses are not adequately recognized. An understanding of the distinct characteristics of ID offenders is crucial during both during the criminal justice process and when forming treatment programs.

## Data Availability Statement

The datasets presented in this article are not readily available because the present study is based upon data from Swedish national registers. Swedish data protection laws and Regional Ethical Review Boards exert joint protection of register data. Study data is consequently not publicly available. Other researchers may however contact Statistics Sweden and the Swedish National Board of Health and Welfare to get access to the different registers included. Requests to access the datasets should be directed to Department of Medical Epidemiology and Biostatistics in Karolinska Institutet, internservice@meb.ki.se.

## Ethics Statement

The studies involving human participants were reviewed and approved by Regional Ethical Review Board in Stockholm (2017/2531-31/5). Written informed consent from the participants' legal guardian/next of kin was not required to participate in this study in accordance with the national legislation and the institutional requirements.

## Author Contributions

HE, TH, and HL designed the study. HL collected the data. HE and QC organized the database. HE, TH, and QC discussed details of data analyses plan. QC performed the statistical analyses. HE analyzed the results supervised by TH, and wrote the first draft of the manuscript. All authors contributed to the conclusions and proposed manuscript revisions, and finally read and approved the submitted version.

## Conflict of Interest

HL has served as a speaker for Evolan Pharma and Shire and has received research grants from Shire, all outside the submitted work. The remaining authors declare that the research was conducted in the absence of any commercial or financial relationships that could be construed as a potential conflict of interest.
